# CUTANEOUS MANIFESTATIONS OF SYSTEMIC LUPUS ERYTHEMATOSUS IN A TERTIARY REFERRAL CENTER

**DOI:** 10.4103/0019-5154.53189

**Published:** 2009

**Authors:** Alakes Kumar Kole, Alakendu Ghosh

**Affiliations:** *From the Institute of Post Graduate Medical Education and Research, Department of Rheumatology and Clinical Immunology*

**Keywords:** *Cutaneous manifestations*, *systemic lupus erythematosus*, *organ involvement*

## Abstract

**Background:**

Systemic lupus erythematosus (SLE) is an autoimmune disease with multiorgan involvement. The skin is the second most commonly affected organ. SLE with skin lesions can produce considerable morbidity resulting from painful skin lesions, alopecia, disfigurement, etc. Skin lesions in patients with lupus may be specific (LE specific) or may be non specific (LE non specific). Acute cutaneous LE (Lupus specific) has a strong association with systemic disease and non-specific skin lesions always indicate disease activity for which patients present to rheumatologists and internists. Therefore, a thorough understanding of the cutaneous manifestations of SLE is essential for most efficient management.

**Aims:**

The aims of this study were to evaluate the patterns and prevalence of skin lesions in patients with SLE and to assess the relationship between skin lesions and other systemic involvement.

**Materials and Methods:**

At the Department of Rheumatology and Clinical Immunology, IPGME&R in Kolkata, 150 patients with SLE fulfilling the clinical and laboratory criteria of the American Rheumatology Association (updated 1982) were examined and followed-up for cutaneous manifestations between January 2002 and January 2007.

**Results:**

Skin lesions were important clinical features. About 45 patients (30%) presented with skin lesions although all patients had skin lesions during the follow-up period. Skin changes noted were as follows: Lupus specific lesions: malar rash in 120 patients (80%), photosensitive dermatitis in 75 patients (50%), generalized maculopapular rash in 40 patients (26.67%), discoid rash in 30 patients (20%), subacute cutaneous lupus erythematosus (SCLE) in 5 patients (3.34%), lupus profundus in 5 patients (3.34%). The lupus non-specific lesions were non-scarring alopecia in 130 patients (86.67%), oral ulcers in 85 patients (56.67%), vasculitic lesions in 50 patients (33.34%), bullous lesions in 15 patients (10%), Raynaud's phenomenon in 10 patients (6.67%), pyoderma gangrenosum in 2 patients (1.34%), erythema multiforme in 10 patients (6.67%), and nail fold infarcts in 2 patients (1.34%); however, mucosal discoid lupus, lichenoid discoid lupus, livedo reticularis, sclerodactyly, etc. were not detected. Patients having lupus-specific skin lesions e.g., malar rash were associated with systemic involvement, whereas those having lupus non-specific skin lesions were associated with disease flare.

**Conclusions:**

Skin lesions in patients with SLE are important disease manifestations and proper understanding is essential for diagnosis and efficient management.

## Introduction

SLE is a heterogeneous autoimmune disease marked by diverse patterns of auto-antibody production with multi-organ involvement. The spectrum of disease ranges from minor organ involvement (e.g., cutaneous lesions) to life-threatening major organ involvement (e.g., renal, nervous system, etc.).

Skin is the second most commonly affected organ after joint involvement and skin lesions are the second most frequent way that this disease presents itself.[1] Skin and mucous membrane are symptomatically involved at some point in over 80% of patients with SLE.[2] Skin lesions in these patients produce considerable morbidity by producing alopecia, scarring lesions, disfigurement, etc. and for these reasons about 45% of patients experience some degree of vocational handicap.[3]

Skin lesions in patients with SLE are classified as those for lupus-specific disease e.g., malar rash, and those for lupus non-specific disease e.g., alopecia (Gilliam classification).[4] There is great variation in incidence, clinical heterogenecity, and severity of disease between different ethnic and racial groups due to environmental, cultural, and genetic variability.[5] Diversity was also noted in the type of skin involvement ranging from classical butterfly rash, discoid lupus to bullae, alopecia, vasculitic rashes, etc.[6]

Cutaneous lesions are important as a diagnostic aid as these account for 4 out of 11 revised ARA criteria for disease classification. Moreover, lupus-specific skin lesions serve primarily as an important diagnostic clue whereas lupus non-specific skin lesions are associated with more active disease and thus require more aggressive therapy and disease monitoring.[7] Thus, a thorough understanding of cutaneous lesions in SLE is critical for efficient diagnosis and management.

## Materials and Methods

SLE patients (n=150) attending the Rheumatology and Immunology Clinic between January 2002 and January 2007 were evaluated and followed-up. The patients were analyzed according to their age, gender, and clinical features with special attention paid to cutaneous manifestations and disease activity (SLE disease activity index). Laboratory investigations included complete blood count, erythrocyte sedimentation rate, urine analysis, anti nuclear antibody, anti-ds DNA, chest X ray, ultra sonography kidney (biopsy), and echocardiography. Exclusion criteria were skin lesions like folliculitis, cellulitis, candidiasis, tinea infections, scabies, drug rash, etc.

## Results

**Figure 1:**
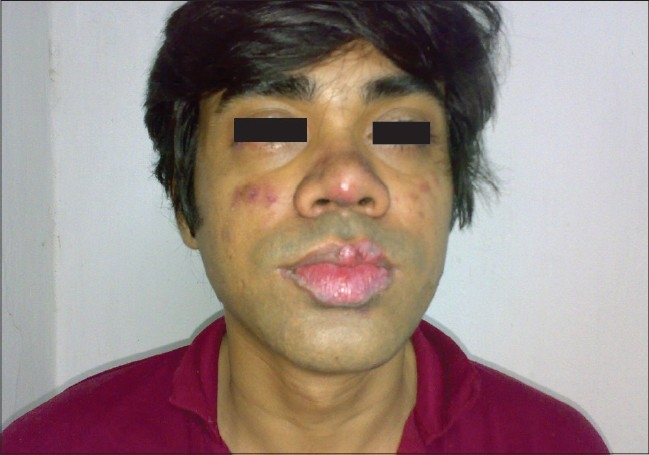
A case of SLE with malar rash and lip DLE

**Figure 2:**
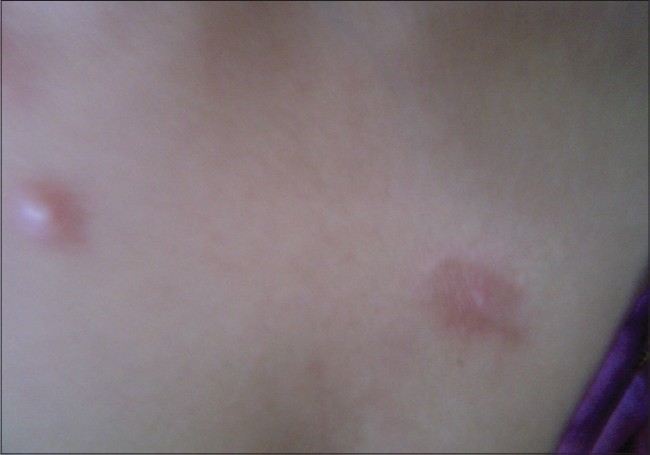
A case of early DLE occupying the sun exposed area

**Figure 3:**
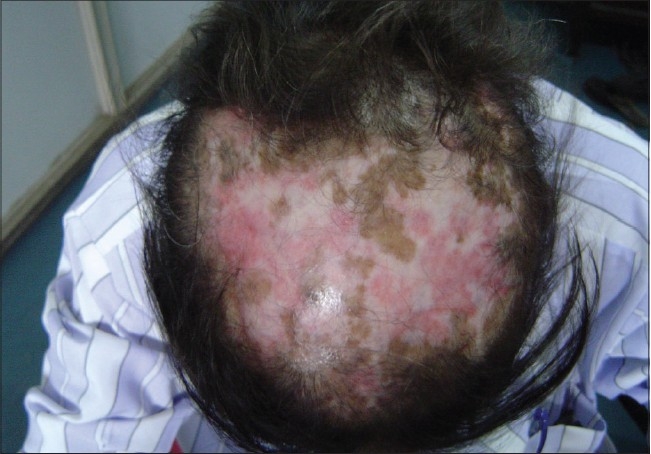
A case of DLE on the scalp with scarring alopecia

Of the 150 patients, 140 (88%) were female and 10 (12%) were male. The female to male ratio was 14:1. The mean age at presentation was 30 years. All patients (100%) developed skin lesions during their follow-up period; although, at the time of presentation only 45 patients (30%) had cutaneous lesions and one third of the patients had acute presentation. The lupus erythematosus-specific lesions were noted as malar rash in 120 patients (80%) [Figure 1], photosensitive dermatitis in 75 patients (50%), generalized maculopapular rash in 40 patients (26.67%), discoid rash in 30 patients (20%) [Figures 2 and 3], subacute cutaneous lupus in 5 patients (3.34%), and lupus profundus in 5 patients (3.34%) but mucosal DLE, lichenoid DLE, and chilblain lupus were not detected.

**Figure 4:**
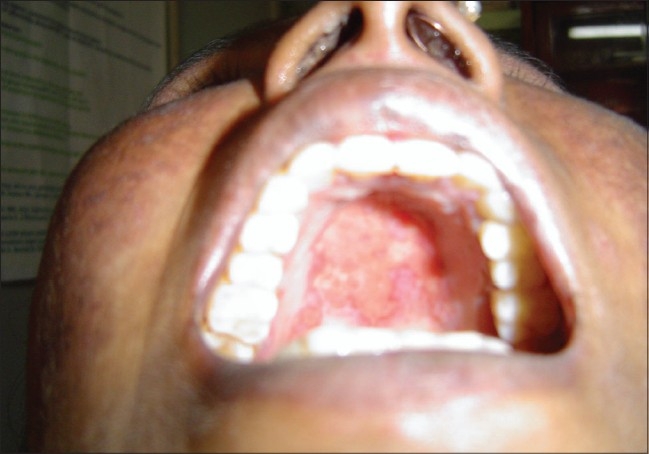
A case of SLE with painless oral ulcers

**Figure 5:**
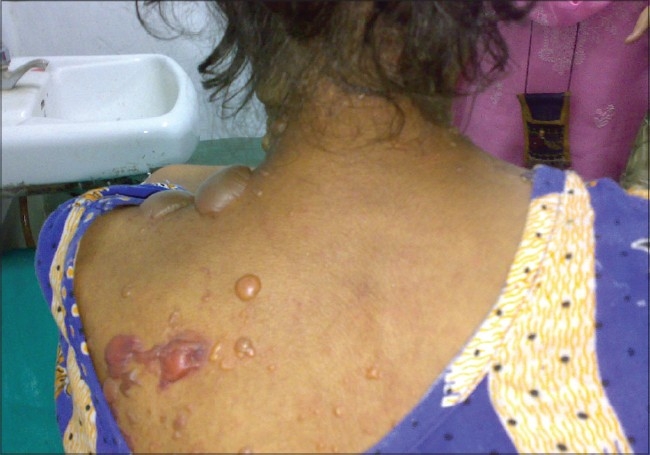
A case of SLE with bullous lesions

**Figure 6:**
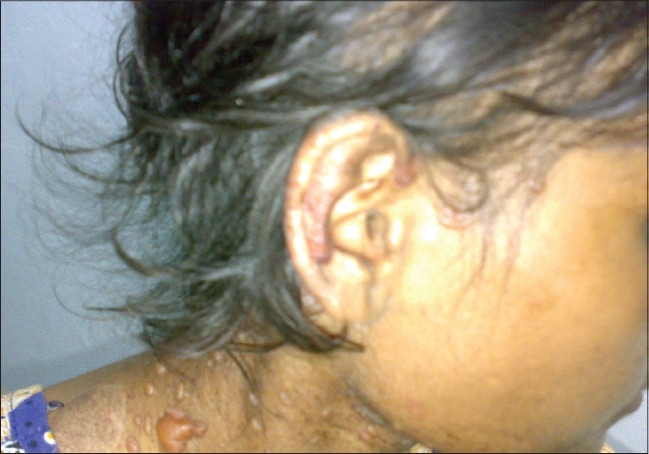
A case of SLE with bullous lesions

**Figure 7:**
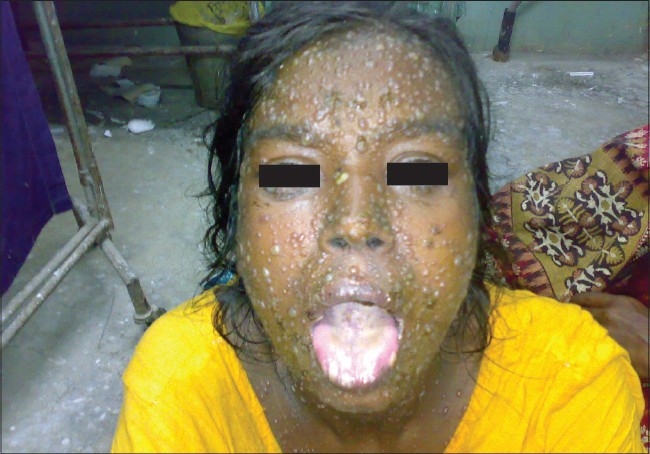
A case of SLE with multiple bullous lesions occupying almost all of the body parts

**Figure 8:**
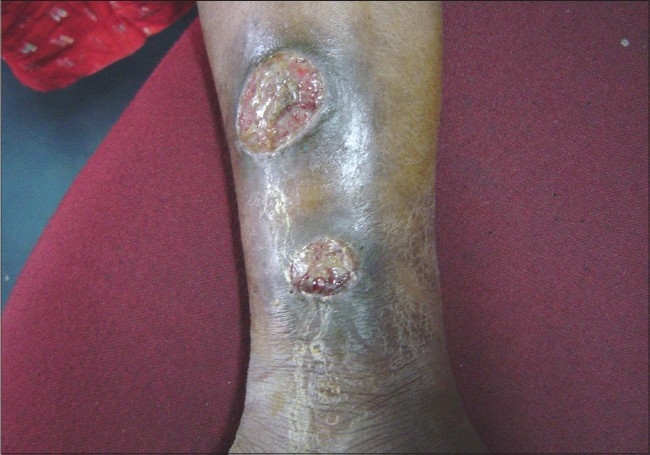
A case of SLE with pyoderma gangrenosum

LE non-specific skin lesions noted were non scarring alopecia in 130 patients (86.67%); scarring alopecia in 10 patients (6.67%); oral ulcers in 85 patients (56.67%) [Figure 4], which were mostly painless; vasculitic lesions in 50 patients (33.34%); bullous lesions in 15 patients (10%) [Figures 5–7] involving the retroauricular region, palms, upper trunk, etc. with frequent relapses; Raynaud's phenomenon in 10 patients (6.67%); erythema multiformae in 10 patients (6.67%); leg ulcers in 10 patients (6.67%); urticaria in 10 patients (6.67%); panniculitis in 2 patients (6.67%); periungual telanangiectasia in 2 patients (1.34%); pyoderma gangrenosum in 2 patients (1.34%) [Figure 8]; and nail-fold infarct in 2 patients (1.34%); but lichen planus, sclerodactyly, livedo-reticularis, erythromelalgia, acanthosis nigricans, calcinosis, facial edema hyperpigmentation, and bluish pigmentation of the nails were not detected.

One important observation in a female patient with SLE was generalized maculopapular rash with subsequent denudation of skin simulating toxic epidermal necrolysis (TEN). She stabilized gradually after three episodes of relapse.

Other system involvements noted were constitutional symptoms in 145 patients (96.67%) in the forms of fever, anorexia, malaise, etc.; polyarthritis in 135 patients (90%); nephritis in 70 patients (46.67%) with active sediments in urine in 30 patients (20%); proteinuria in 40 patients (26.67%); cardio-pulmonary involvement in 20 patients (13.34%); hematological disturbances in 125 patients (83.34%); neuro-psychiatric involvement in 110 patients (73.34%); and gastro intestinal involvement in 70 patients (46.67%). Lymphadenopathy (20%) mostly showed reactive hyperplasia on histology.

All patients were ANA-positive (100%) and anti ds-DNA was detected in 83.34% of the cases [Tables 1–4].

**Table 1:** LE-specific skin lesions

	Malar rash	Photosensitive dermatities	Generalized maculopapular rash	SCLE	DLE	Lupus profundus
No. of patients	120	75	40	5	30	5
Percentage	80	50	26.67	3.34	20	3.34

SCLE-Subacute cutaneous lupus erythematosus; DLE-Discoid lupus erythematosus

**Table 2a:** LE non-specific skin lesions

	Alopecia (non scarring)	Oral ulcers	Vasculitis lesions	Bullous lesions	Erythema multiforme	Leg ulcer
No. of patients	130	85	50	15	10	10
Percentage	86.67	56.67	33.34	10	6.67	6.67

**Table 2b:** LE non-specific skin lesions

	Raynaud's phenomenon	Urticaria	Nail fold infarct	Periungual telangiectasia	Thrombophlebitis
No. of patients	10	10	2	2	15
Percentage	6.67	6.67	1.34	1.34	10

**Table 3:** Cumulative incidence of clinical and immunological manifestations of in other studies

Clinical manifestations	Dubos (1974) *n* = 520 USA (%)	Malaviya (1985) *n* = 101 North India (%)	Vaidya (1997) *n* = 220 West India (%)	Present study *n* = 150 West Bengal (Kolkata) (%)
Dermatologic	NA	85	NA	100 (*n* = 150)
Malar rash	NA	NA	53.18	80 (*n* = 120)
Discoid lupus	NA	NA	NA	20 (*n* = 30)
Photosensitivity	43	NA	9.55	50 (*n* = 75)
Oral ulcer	9.1	64	NA	56.67 (*n* = 85)
Alopecia	21	82	NA	86.67 (*n* = 130)
Raynaud's phenomenon	18.4	32	15.5	6.67 (*n* = 10)
Renal involvement	46.1	73	35	46.67 (*n* = 70)
Pulmonary	45	17	15.5	13.34 (*n* = 20)
Cardiovascular	30.5	5	11.8	13.34 (*n* = 20)
Neuropsychiatric manifestations	25.5	15	25.5	73.34 (*n* = 110)

**Table 4:** Other system involvement

	Constitutional symptoms	Musculo skeletal (arthritis)	Hemato-logical	Nervous system	Gastro intestinal	Renal	Lymphadenopathy
No. of patients	145	135	125	110	110	70	30
Percentage	96.67	90	90	73.34	73.34	46.67	20

## Discussion

In the present study, the patients’ age ranged from 10-39 years. The median age for disease onset was 25 years. Masi, *et al.*[8] observed a median age of disease onset of 31 years whereas from India, Malaviya, *et al.*[9] noted a median age of disease onset of 24 years. The peak incidence was seen in the 3^rd^ decade in both series.

In this study, the female to male ratio was 14:1 but Malaviya, *et al*.[9] from India reported a female to male ratio of 8:1.

In the present study, cutaneous involvement was the most common feature (73.34%) in the disease spectrum. Cutaneous lesions were the initial presentation in 25% of the cases as reported by Waston[10]; whereas, Feng, *et al.* and Malaviya, *et al.* reported arthritis as an initial manifestation in 44% and 57% of the patients, respectively.[11,9] Constitutional systems (e.g., fever, weight loss, etc.) was the second most common presentation in this study.

Among the LE-specific cutaneous lesions, malar rash was the most common lesion (80%) noted in this study; whereas Wysenbeek, *et al.*[12] and Vaidya, *et al.*[13] from western India reported malar rash in 49% and 53.18% of the patients, respectively. Lesions of discoid lupus was considerably lower (20%) in this study and more or less corroborated with the studies previously conducted by Kapadia[14] and Wysenbeek.[12]

Diffuse maculopapular rash was noted in 26.67% of the cases in contrast to 59% of the cases as reported by Wysenbeekn, *et al.* Lesions of subacute cutaneous lupus were detected in this study in 3.34% of the cases; whereas, Wysenbeek, *et al.* reported the lesions in 13% of the cases. Mucosal DLE, chilblain lupus, etc. were not detected in this study.

Among LE non-specific, skin lesions non-scarring diffuse alopecia was more frequent (86.67%) as compared to 57% noted by Wysenbeek[12] and 82% by Malaviya.[9] Oral ulcers were seen in this study in 56.67% of the cases as compared to 9.1% and 64% reported by Dubois[15] and Malaviya,[9] respectively. Raynaud's phenomenon is a less common skin lesion in SLE. In this study, we had seen this in 6.67% of the cases, whereas Malaviya, *et al.* from north India and Vaidya, *et al*. from Western India noted Raynaud's phenomenon in 32% and 15.5% of the cases, respectively.[9,13] This variation may be attributable to the climatic condition of that particular region. We had noted two cases of pyoderma gangrenosum in the patients; it is a rare skin lesion, and may be the initial presentation of the disease.[16] Urticaria-like skin lesions are very unusual in patients suffering from SLE[17] but we had noted such lesions in 6.67% of the cases. Dubois mentioned that development of urticaria in a patient with SLE should lead the physician to carefully evaluate that patient for active systemic disease.[18] Bullous lesions are rare blistering conditions occurring in less than 5% of patients with SLE in isolation or in combination with other skin lesions[19] but in this study, 10% of the patients had such lesions.

Bluish discoloration of the nails as noticed commonly by Kapadia, *et al.*[14] was not seen in this study. Livedo reticularis, sclerodactyly, and lichen planus were not observed in this study, which also closely matched the results of the study by Watson, *et al*.[10]

In some cases, skin lesions may be associated with the involvement of other organs. Vasculitic skin lesions in some cases are associated with neuropsychiatric manifestations of lupus[20] but in this study, patients having such lesions were devoid of any overt neuropsychiatric features. In this study, patients having bullous skin lesions had systemic flares that were also reported previously by Malcangi,*et al*.[21] An association between concomittant lupus nephritis and bullous lesions had been documented by Ng, *et al.*[22] but no such association has been documented in this study.

In our study group, patients with LE non-specific skin lesions, specially generalized maculopapular vasculitic lesions, and diffuse non-scarring alopecia were associated with more active disease. This was also reported previously by Zeevi, *et al.*[23] Another interesting finding in this study was that patients with cutaneous lesions had significantly more lymphadenopathy (20%); this was also previously reported by Arjeh, *et al*.[12]

Other systemic involvement included constitutional symptoms (96.67%) such as fever, malaise, musculo skelelal (90%), hematological (83.34%) changes.

## Conclusions

SLE is a multisystem disorder in which cutaneous manifestations can yield valuable diagnostic (e.g., LE-specific skin lesions) as well as prognostic (e.g., LE non specific skin lesions - as these are associated with disease activity) information. Skin lesions are responsible for increased morbidity. So proper understanding regarding skin lesions of SLE will be helpful for the disease diagnosis and efficient management of patients with lupus. For this reason, more interaction between rheumatologists and dermatologists will lead to more specific diagnosis of cutaneous lesions in patients with SLE.
